# Development of an enzyme-linked immunosorbent assay for Keap1-Nrf2 interaction inhibitors identification

**DOI:** 10.1016/j.redox.2020.101573

**Published:** 2020-05-12

**Authors:** Yan Wang, Chu-Ying Xiao, Huang-Quan Lin, Jian-Shu Hu, Tsz-Ming Ip, David Chi-Cheong Wan

**Affiliations:** a School of Biomedical Sciences, Faculty of Medicine, The Chinese University of Hong Kong, Shatin, Hong Kong SAR, China; b Center for Translation Medicine Research and Development, Institute of Biomedical and Health Engineering, Shenzhen Institutes of Advanced Technology, The Chinese Academy of Sciences, Shenzhen, 518055, China; c Shenzhen Research Institute, The Chinese University of Hong Kong, Shenzhen, 518057, China

**Keywords:** Keap1-Nrf2 interaction inhibitor, ELISA, FDA approved Drugs

## Abstract

Development of Keap1–Nrf2 interaction inhibitors is a promising strategy for the discovery of therapeutic agents against oxidative stress-mediated diseases. Two motifs of Nrf2, ETGE and DLG motif, are responsible for Keap1-Nrf2 binding. Previously, ETGE peptide or ETGE-derived peptide-based approaches were used to detect Keap1-Nrf2 interaction; however, these approaches are not able to monitor Keap1-DLG motif binding. We first report here a novel Enzyme-linked Immunosorbent Assay (ELISA) approach to detect the protein-protein interaction of full length Keap1 and Nrf2. In our assay, the test compounds can target either ETGE or DLG binding site, therefore facilitating the exploration of diverse Keap1-Nrf2 inhibitors. Three FDA-approved drugs, zafirlukast, dutasteride and ketoconazole, were found to inhibit the Keap1-Nrf2 interaction with IC_50_ of 5.87, 2.81 and 1.67 μM, respectively. Additionally, these three drugs also activated Nrf2 pathway in neuroblasts and lipopolysaccharide (LPS)-challenged mice. The results presented here indicate that the ELISA approach has the capacity to identify Keap1-Nrf2 inhibitors.

## Highlights

• We established a novel Enzyme-linked Immunosorbent Assay for Keap1-Nrf2 interaction inhibitors identification.
• This ELISA is the first approach to detect the protein-protein interaction of full length Keap1 and Nrf2.
• In this assay, the test compounds can target either ETGE or DLG binding site, therefore facilitating the exploration of diverse Keap1-Nrf2 inhibitors.
• Zafirlukast, dutasteride and ketoconazole have been identified as inhibitors of the Keap1-Nrf2 interaction.

## Graphical abstract

**Figure:**
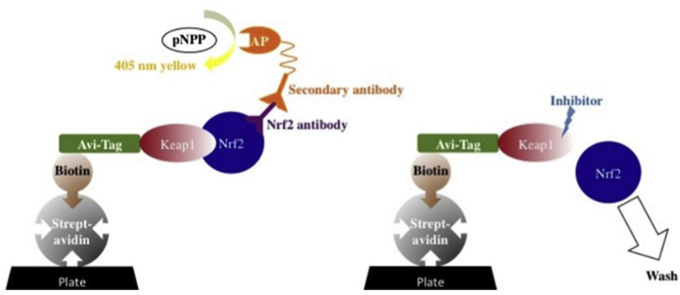

## 1 Introduction

**Fig. 1:**
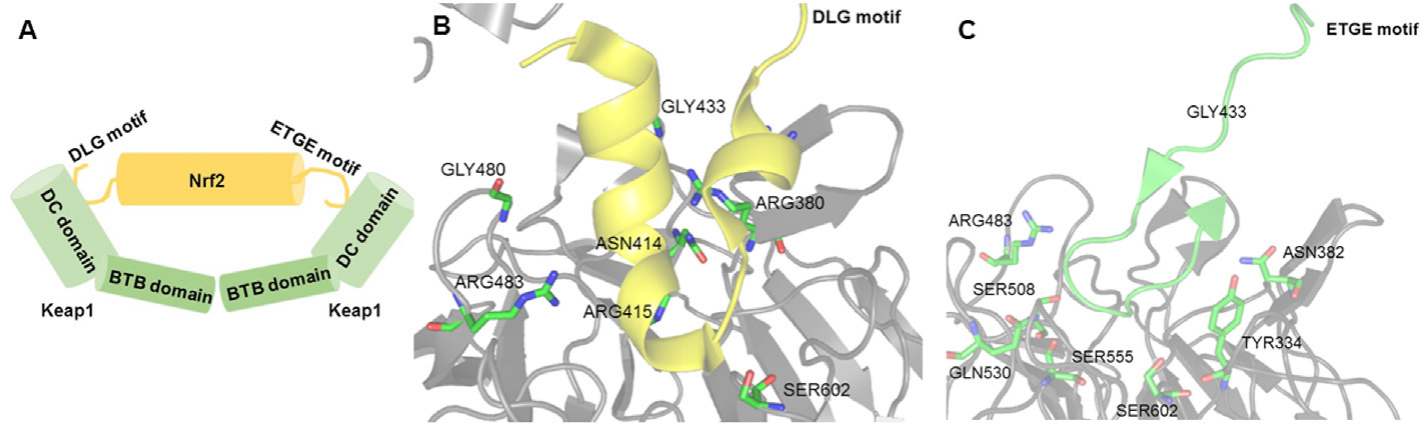
**Binding of Nrf2 to Keap1.** (A)Schematic binding mode. (B) Selected side-chain interactions in the DLG motif complex with mouse Keap1 (PDB 3WN7). DLG peptide are colored yellow; Keap1 protein are colored gray while the key residues for DLG binding are highlighted in green stick. (C) Selected side-chain interactions in the ETGE motif complex with mouse Keap1 (PDB 2FLU). ETGE peptide are colored green; Keap1 protein are colored gray while the key residues for ETGE binding are highlighted in green stick. (For interpretation of the references to color in this figure legend, the reader is referred to the Web version of this article.)

Oxidative stress has been demonstrated involving into many pathophysiological processes that would lead to diseases including cancer, diabetes, cardiovascular and neurodegenerative diseases [1]. In order to defense oxidative stress, cells undergo anti-oxidative processes [1]. Antioxidant defense system contains several key enzymes, including NADPH: quinone oxidoreductase 1 (NQO1), heme oxygenase-1 (HO-1), superoxide dismutase (SOD), and glutathione S-transferase (GST). These enzymes are mainly regulated by Kelch-like ECH-associated protein 1 (Keap1) and nuclear factor erythroid 2-related factor 2 (Nrf2) [2]. Under normal conditions, Nrf2 is captured by Keap1 via its Neh2 domain. When the cell is challenged with oxidative stress, there is a conformation change of Keap1 through dissociation of Nrf2, followed by Nrf2 nuclear translocation to trigger the transcriptional activation of NQO1 and HO-1 [2]. Keap1 acts as the suppressor of Nrf2 (Fig. 1A). The BTB domain has an association with Cullin3 (Cul3) that further mediates the ubiquitination of Nrf2 [3]. The DRG and CTR domain (together so called DC domain) is the region that is associated with Nrf2 [3]. Nrf2 is a basic region leucine ziprper (bZip) transcription factor of the cap “n” collar subfamily. It contains six domains: Neh1 to Neh6. Two motifs, ETGE motif and DLG motif locating on Neh2 domain are responsible for Keap1-Nrf2 binding (Fig. 1B and C) [4]. Given that the activation of Nrf2 consequently upregulates a series of cytoprotective genes to protect cells from oxidative stress-induced cell damage, Keap1–Nrf2 protein-protein interaction (PPI) inhibitors has been proposed as an important approach for the treatment of chronic oxidative and inflammatory stress [5].

To evaluate Keap1-Nrf2 PPI inhibitors, several biochemical assays have been developed, including competitive fluorescence polarization (FP) assay, SPR-based competition assay, fluorescence resonance energy transfer (FRET) assay, tracer displacement assay and differential scanning fluorimetry (DSF) assay [5]. Among these assays, two fluorescence-based approaches are most popular for the Keap1-Nrf2 inhibitor screening. In FP assay, the Keap1 protein and fluorescently labeled Nrf2 peptide are incubated with test compounds [6]. The free fluorescently labeled Nrf2 peptide exhibits low fluorescent polarization whereas bound Nrf2 peptide to Keap1 significantly enhances fluorescent polarization. This change in fluorescent polarization can then be measured by using a fluorescence reader [6]. Compounds that reduce the fluorescent polarization in FP assay are identified as Keap1-Nrf2 inhibitors. Additionally, the basis of FRET assay is the binding of a YFP-conjugated Keap1 to a CFP-conjugated Nrf2-derived 16-mer peptide containing a highly conserved “ETGE” motif [7]. FRET is observed as a decreased emission at 475 nm and an increased emission at 527 nm, which can be abolished by Keap1-Nrf2 inhibitors [7].

Both FP and FRET assays utilize fluorescent label strategy and avoid the use of separation steps, leading to its popular application in HTS assay development [8]. However, either FP or FRET is relatively sensitive to autofluorescence of test compounds [9]. An alternative assay should be available to validate the bioactivity of hits that are filtered by FP or FRET screening. Interestingly, both of these two approaches employed Nrf2 peptide or Nrf2-derived peptide to monitor Keap1-Nrf2 ETGE peptide disruption (Fig. 1C). The full length of Nrf2 should be used in the alternative assay to determine whether hits can block the protein-protein interaction between full length Keap1 and Nrf2.

The enzyme-linked immunosorbent assay (ELSIA) is a binding analysis approach, which can monitor PPI in solution [10]. For example, the inhibitors of Myc/Max dimerization were filtered by FRET-based HTS assay; the hits were then validated by ELISA [11]. Additionally, the inhibition of MDM2–p53 protein–protein interaction by small molecules was also evaluated by ELISA [12]. Therefore in this study, we established a novel ELISA-based approach for Keap1-Nrf2 inhibitors identification in this study.

## 2 Result and discussion

### 2.1 ELISA format approach development

The blueprint of plasmid construction is shown in Fig. S1A. Keap1 with Avi-tag and BirA ligase were inserted into pCOLADuet-1 vector. Keap1 was probed by both alkaline phosphatase (AP) conjugated streptavidin and Keap1 antibody that showed single band with same molecular weight at 70 kDa (Fig. S1B). The purified Keap1 was labeled with biotin successfully because it can be detected by the streptavidin-conjugated antibody. Human Nrf2 has a predicted molecular mass of 66 kDa [13]. However, our immunoblots showed that single band at about 110 kDa in Fig. S1C. Our Western blot result shows a single band probed by the Nrf2 antibody before and after purification. The migration of molecular weight is likely due to an abundance of acidic residues in Nrf2 [13].

**Fig. 2:**
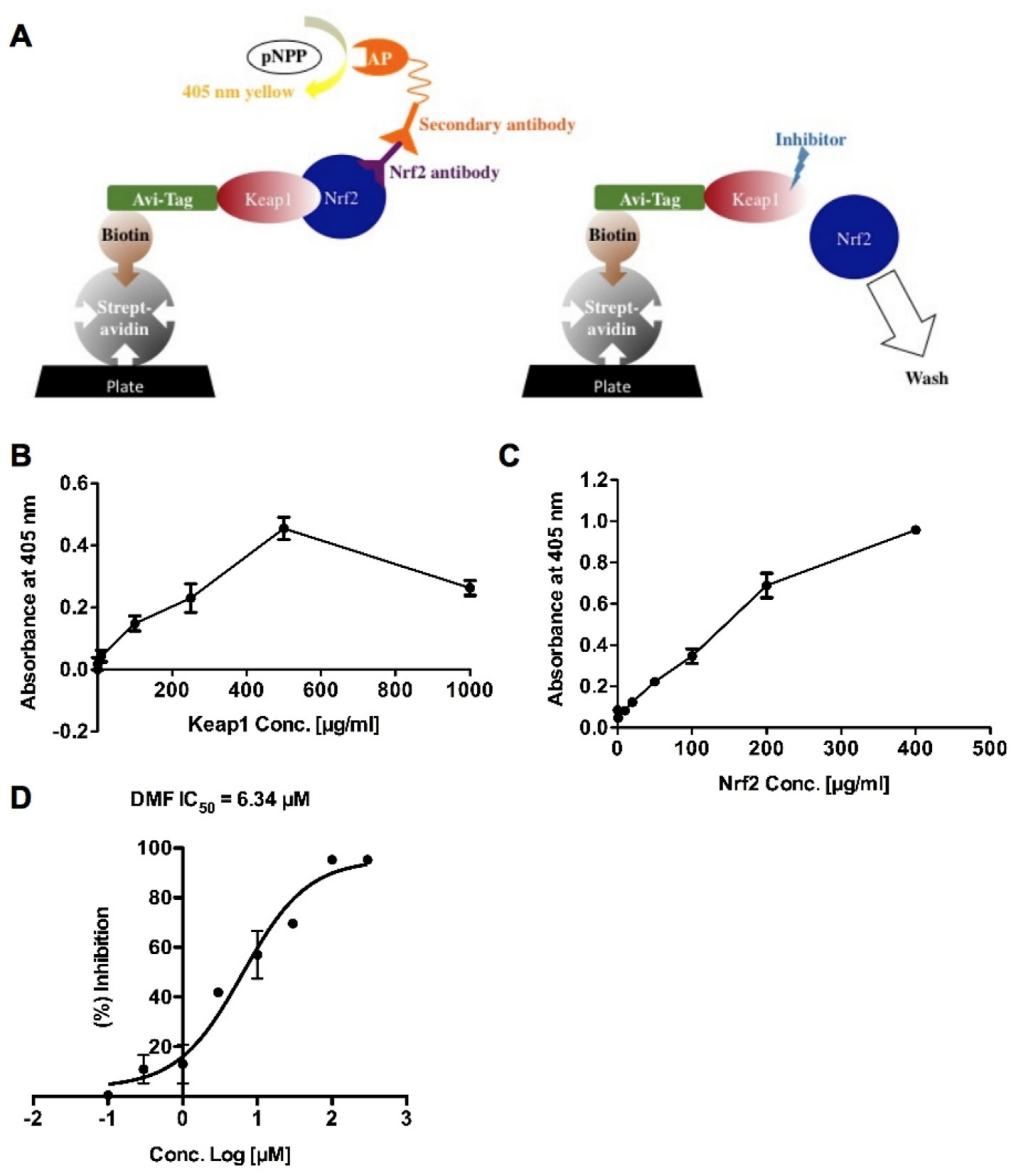
**Establish a novel ELISA format assay to evaluate inhibitory effects of chemicals on Keap1-Nrf2 interaction.** (A) Schematic description of ELISA format assay for Keap1-Nrf2 small-molecule inhibitors screening. (B) To determine the binding ability of biotinylated Keap1 against the streptavidin-coated plate, serial dilution of biotinylated Keap1 was added to the plate. The plate was incubated at room temperature for 30 min and washed three times with plate washing buffer. The streptavidin-binding Keap1 was quantified by streptavidin conjugated alkaline phosphatase. (C) To determine the binding ability of Keap1 against Nrf2, serial dilution of His-tag Nrf2 was incubated with Keap1 for 30 min and washed three times with plate washing buffer. The Keap1-binding Nrf2 was probed by Nrf2 antibody and quantified by alkaline phosphatase conjugated secondary antibody. (D) The inhibitory effect of DMF on Keap1-Nrf2 interaction was analyzed by ELISA format assay.

We then designed an ELISA format to detect the protein-protein interaction of Keap1 and Nrf2, and a schematic description of this assay is illustrated in Fig. 2A. Keap1 protein was labeled with an Avi-tag allowing ligation of biotin. The biotinylated Keap1 bound to a streptavidin-coated plate through the biotin-streptavidin interaction. Then full length of Nrf2 was added to the plate and captured by Keap1. The captured Nrf2 was probed with the Nrf2 primary antibody. Then the Nrf2 antibody was probed with AP-conjugated secondary antibody and detected with AP substrate pNPP that produces a yellow product that absorbs light at 405 nm. When adding Keap1-Nrf2 PPI inhibitor to the system, Nrf2 is supposed to escape from Keap1 and could be washed off so that the absorbance at 405 nm would be decreased.

The absorbance at 405 nm was increased as the Keap1 concentration increased (up to 500 μg/mL, Fig. 2B). Additionally, the concentrations of Nrf2 were also positively correlated with the absorbance at 405 nm (Fig. 2C). Therefore 250 μg/mL of Keap1 and 250 μg/mL of Nrf2 were selected for our assay development. We used dimethylformamide (DMF) as a positive control to validate the new ELISA format. (Fig. 2D). DMF was reported to activate Nrf2 pathway in cells at low micormolar dose [14]. In our assay, DMF inhibited Keap1-Nrf2 interaction with IC_50_ of 6.34 μM (Fig. 2D).

Currently FP and FRET assays are two common approaches for evaluating Keap1-Nrf2 PPI inhibitors [5]. Both FP and FRET assays utilize fluorescent label strategy which can be applied to HTS assay development. 9-mer Nrf2 peptide amide was labeled with FITC probe in FP assay while 16-mer Nrf2-derived peptide was conjugated to CFP in FRET assay [6,7]. However, either FITC or CFP is relatively sensitive to autofluorescence of test compounds. Additionally, these peptides in fluorescence-based assays mimic ETGE motif that docks with Keap1-DC (Fig. 1C), indicating that these assays monitor Keap1-Nrf2 ETGE peptide disruption [6,7]. However, Nrf2 contains two motifs, ETGE and DLG locating on Neh2 domain for Keap1-Nrf2 binding (Fig. 1B and C) [4]. In our novel assay, full-length Nrf2 was used. It suggested that our assay can identify the inhibitors targeting either ETGE binding site (Fig. 1C) or DLG binding site (Fig. 1B). The ELISA approach was selected for our assay development to avoid autofluorescence interference produced by test compounds.

### 2.2 Three FDA-approved drugs as novel inhibitors of Keap1-Nrf2 interaction

Structure-based virtual screening has been utilized to identify Keap1–Nrf2 PPI inhibitors from SPECS database, and several hits with low micromolar *K*_d_ were identified [15]. Apart from synthetic compound library, U.S. Food and Drug Administration (FDA)-approved drugs provide another good pool for hits screening [16]. In this study, we employed molecular docking screening to identify novel Keap1-Nrf2 PPI inhibitors from an FDA-approved drug database of ~1500 compounds [17]. The top 20 candidates from FDA-approved drugs screening are summarized in Table S1. Seven drugs out of the top 20 candidates were selected for anti-Keap1-Nrf2 PPI activity test using both ELISA and FRET assays. The structures of these seven drugs are shown in Fig. S2.

**Fig. 3:**
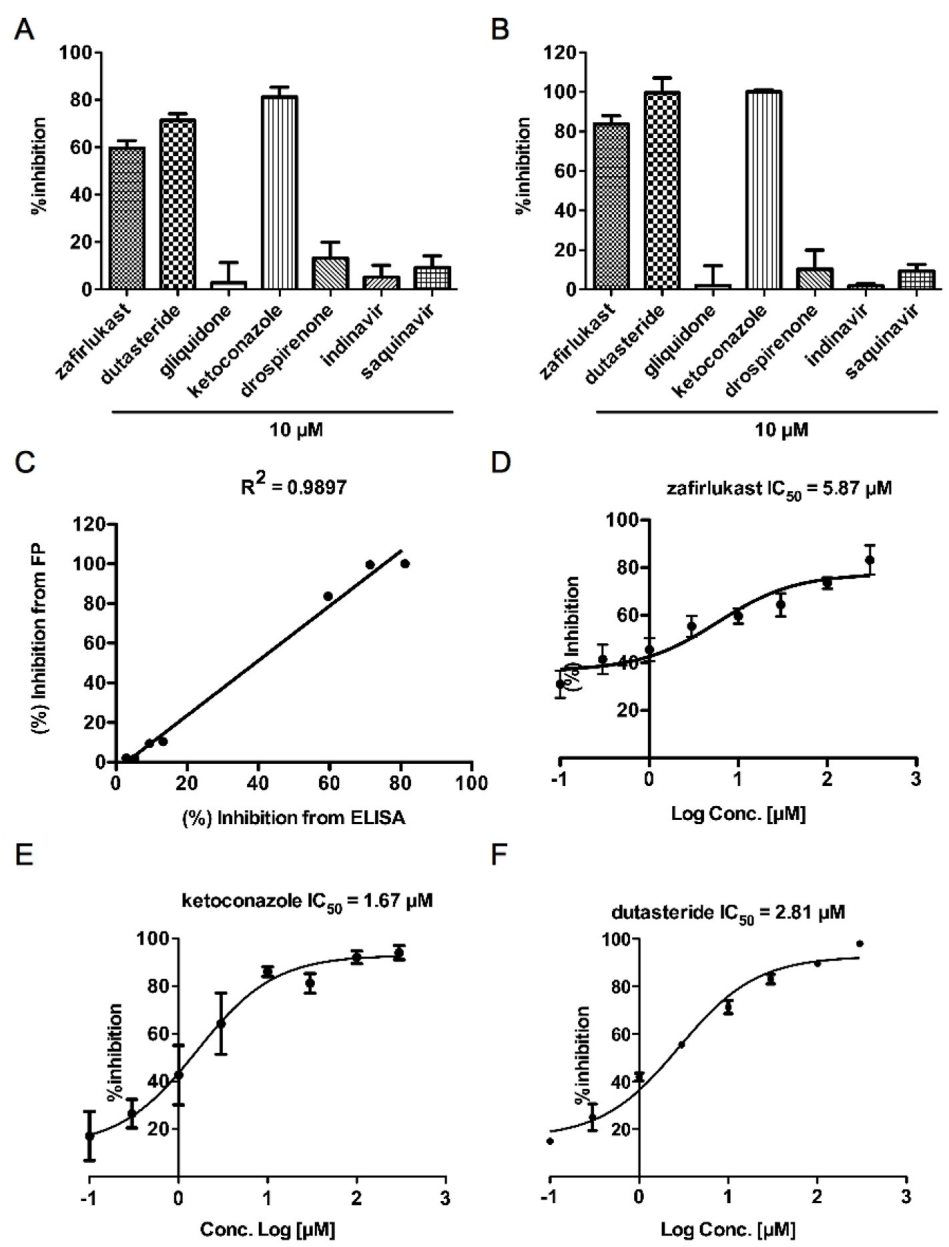
**Three FDA-approved drugs are identified as novel inhibitors of Keap1-Nrf2 interaction with IC**_**50**_**at low micro molar.** (A) The inhibitory effects of selected drugs on Keap1-Nrf2 interaction were analyzed by ELISA format assay at 10 μM. (B) The inhibitory effects of selected drugs on Keap1-Nrf2 interaction were analyzed by FP format assay at 10 μM. (C) There was a strong correlation between the inhibitory response in FP assay and the inhibitory response in ELISA format assay (R^2^ = 0.9897). Dose–response inhibition of zafirlukast (D), ketoconazole (E), and dutasteride (F) on Keap1-Nrf2 interaction was evaluated by ELISA format assay. The IC_50_ values were calculated using the inhibitor dose–response function in Prism 5.

Seven drugs were tested their inhibitory activity of Keap1-Nrf2 PPI using our novel ELISA format assay as well as FP assay. In our ELISA test, the percentage inhibitions of gliquidone, drospirenone, indinavir and saquinavir were less than 20% (Fig. 3A). However, three drugs (zafirlukase, dutasteride and ketoconazole) exhibited potent inhibitory effects with more than 60% inhibition at 10 μM (Fig. 3A). In the fluorescent polarization assay, zafirlukast inhibitory activity was up to 80% while inhibitory activities of dutasteride and ketoconzale were up to 100% (Fig. 3B). Other fours compounds did not block Keap1-Nrf2 interaction at 10 μM (Fig. 3B). The strong correlation (r^2^ = 0.9897) between inhibitory response of FP assay and ELISA format assay is another evidence showing our ELISA format assay is reliable (Fig. 3C). Dose-response inhibition of three candidates was evaluated by an ELISA. The IC_50_ of zafirlukast was 5.87 μM, ketoconazole was 1.67 μM and dutasteride was 2.81 μM (Fig. 3D, E, and F).

### 2.3 Newly identified Keap1-Nrf2 PPI inhibitors activated Nrf2 pathway in cells

When Nrf2 is activated, cytoplasmic-nuclear translocation of Nrf2 should be observed. To further validate the mechanism of action, Western blot was used to characterize the subcellular distribution of Nrf2 protein in SH-SY5Y and PC12 cells. Rat PC12 and human SH-SY5Y cells are widely used in antioxidant studies due to their oxidative stress sensitivity. When treated cells with 10 μM of candidates, the nuclear Nrf2 level increased and in contrast cytoplasm Nrf2 level decreased compared to controls in both SH-SY5Y cells and PC12 cells (Fig. S3A). It suggested that the three selected drugs induced Nrf2 nuclear translocation. In order to explore whether drugs have effects on Nrf2 mRNA expression level, total mRNA was collected and Nrf2 mRNA level was measured (Fig. S3B). We did not detect a significant change in Nrf2 mRNA level, indicating that the drugs did not influence Nrf2 mRNA expression.

**Fig. 4:**
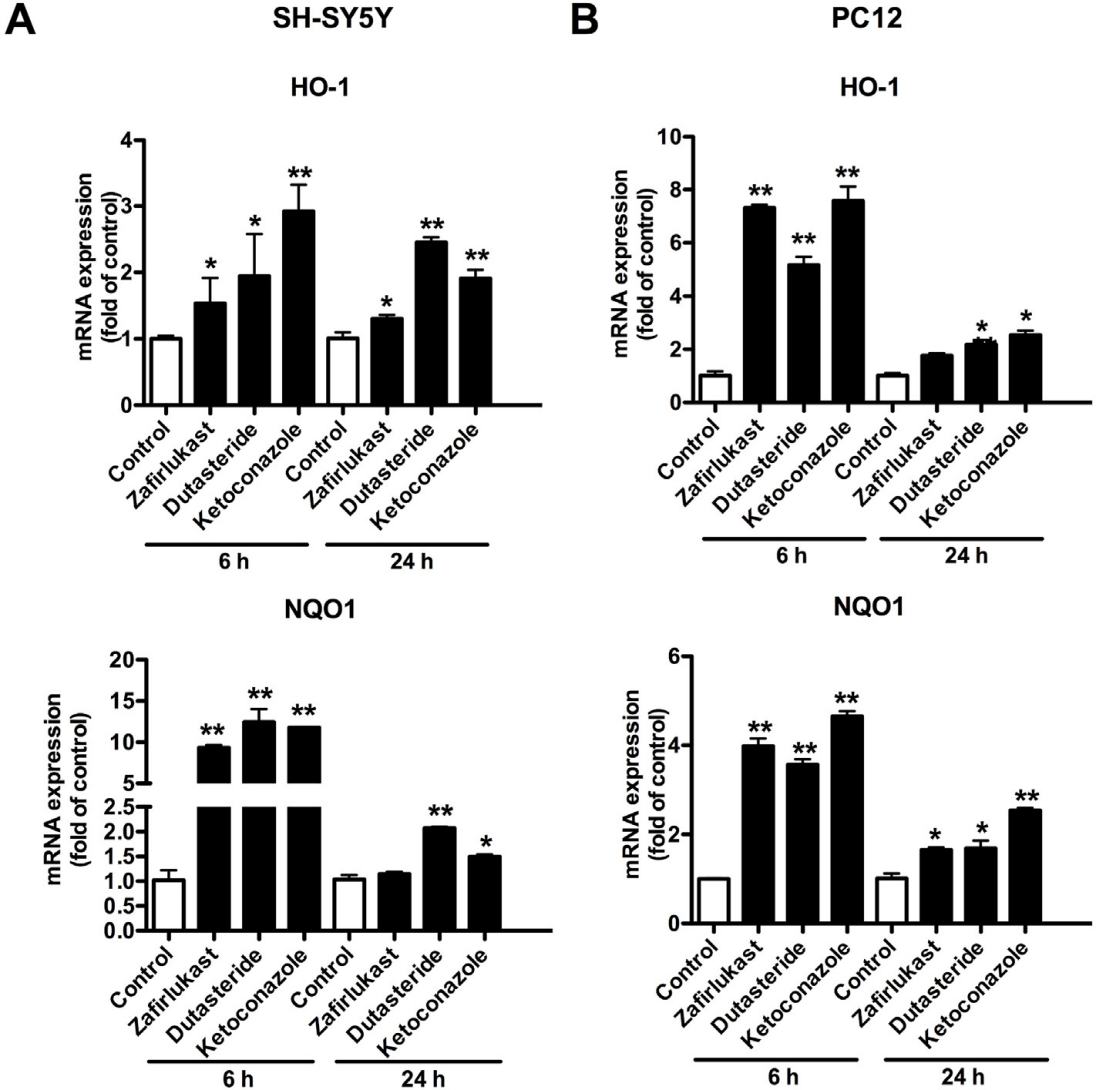
**Three FDA-approved drugs upregulate Nrf2-mediated gene expression in both SH-SY5Y cells and PC12 cells.** SH-SY5Y cells (A) or PC12 cells (B) were treated with DMSO or test drugs (10 μM) for 6 h or 24 h. Then the cells were harvested, the total mRNA was collected for reverse transcription, and quantitative PCR measured. Results were repeated at least three times from three independent experiments and presented as mean ± SD, n = 3. The results were statistically analyzed by one-way ANOVA test. *P < 0.05 compared with control cells; **P < 0.01 compared with control cells.

Nrf2 activates gene transcription through the antioxidant-responsive element (ARE) [18]. ARE was found in the promoters of genes encoding the two major detoxication enzymes, HO-1 and NQO1 [18]. To determine whether the compounds activate ARE by Nrf2, qPCR was carried out to quantify the mRNA levels of HO-1 and NQO-1 in SH-SY5Y and PC12 cells. Both HO-1 and NQO1 mRNA expression levels were significantly enhanced by three test compounds at 6 h (Fig. 4). However, when treating cells for 24 h, the mRNA expression level of Nrf2 downstream genes were relatively declined (Fig. 4). The mRNA expression level decreasing for long time treated cells could be caused by the Nrf2 negatively regulation loop [19].

The upregulation of HO-1 and NQO1 expression was found to protect cells against the oxidative-stress cellular injury [2]. Here we used H_2_O_2_ to induce cell death [20]. Four hours treatment of 250 μM H_2_O_2_ decreased cell viability significantly in both SH-SY5Y and PC12 cells (p < 0.005, Fig. S3B). When treating cells with 10 μM zafirlukase, dutasteride or ketoconazole for 24 h, cell viability was not changed (Fig. S4A). In order to evaluate the antioxidative effects of three drugs, both SH-SY5Y and PC12 cells were treated with drugs for 24 h following 4 h treatment of 250 μM H_2_O_2_. As showed in Fig. S4B, in SH-SY5Y cells, zafirlukast and dustasteride can improve cell viability for 10% (P < 0.05) and ketoconazole can improve cell viability for more than 20% significantly (P < 0.01). Meanwhile in PC12 cells, zafirlukast, dutasteride and ketoconazole can all increase cell viability from 20% to about 60% significantly (P < 0.005).

Taken together, zafirlukase, dutasteride and ketoconazole promoted Nrf2 nuclear translocation and upregulated Nrf2 downstream gene expression, therefore protecting cells against oxidative-stress cellular injury.

### 2.4 Newly identified Keap1-Nrf2 inhibitors relieved mouse inflammatory responses induced by LPS challenge

After the validation of the potency of newly identified Keap1-Nrf2 PPI inhibitors in the cell-based experiments, the *in vivo* anti-inflammatory effects of these three drugs in the context of the LPS challenge was evaluated. LPS was reported to lead to both MyD88-dependent early phase NF-κB transcription of pro-inflammatory cytokines, such as TNF-α, and IL-6 and IL12 [21]. LPS-challenged mice exhibited the inflammatory response which has been successfully alleviated by Keap1-Nrf2 PPI inhibitors [22]. The pro-inflammatory cytokines are characterized as biomarkers for inflammation in LPS-induced animal models, and these inflammatory cytokines also lead to the inflammatory damage [23]. Therefore we selected the circulating inflammatory cytokines as biomarkers for monitoring anti-inflammatory effects. The mice (except the blank control group and LPS group) will be treated with dexamethasone or Keap1-Nrf2 PPI inhibitors by intragastric administration for 5 days (day 1, 2, 3, 4 and 5) beginning at 12–16 weeks old. All mice (except the blank control group) will be challenged with LPS by intraperitoneal injection at day 5. 5 h after LPS challenging, all mice were sacrifised by overdose anesthesia and blood were collected. IL-6, IL-12 p70 and TNF-α in serum samples were measured by ELISA kit.

As shown in Fig. S5, both high dose and low dose Keap1-Nrf2 PPI inhibitors significantly reduced the levels of pro-inflammatory cytokines, including TNF-α, IL-6, and IL-12, relative to LPS-challenged mice. Furthermore, three drugs had comparable effects on IL-6 and TNF-α at the same concentration as the positive control dexamethasone (10 mg/kg/day). In general, these results suggested that three new Keap1-Nrf2 PPI inhibitors pretreatment can reduce inflammatory cytokines and confer protection against LPS challenge.

## 3 Conclusion

Together, we first report here a novel ELISA approach to identify compounds that inhibit PPI of full length Keap1 and Nrf2, therefore providing a secondary assay for Keap1-Nrf2 PPI inhibitors development. We summarized the advantage and disadvantage of ELISA and other assays in Table S4. Basically, ELISA could avoid high background noise which is always interrupts fluorescent signal in FP and FRET assays. Additionally, Keap1 binds to Nrf2 via two binding spots, Keap1-ETEG binding site and Keap1-DLG binding site. ELISA could identify both the Keap1-DLG binding inhibitors and Keap1-ETEG binding inhibitors. Conversely, FP or FRET assays only identify Keap1-ETEG binding inhibitors. Our ELISA screening could facilitate the exploration of diverse Keap1-Nrf2 inhibitors. Three of FDA-approved drugs have been identified as Keap1-Nrf2 PPI inhibitors with low-micromolar IC_50_ values using our ELISA approach. Apart from the direct binding assay, these three drugs also activated Nrf2 pathway in SH-SY5Y and PC 12 cells. Additionally, these three drugs attenuated LPS-induced inflammation in mice, as would be expected for a compound that targets Keap1-Nrf2 PPI. We anticipate that zafirlukast, dutasteride and ketoconazole could be further explored to act as novel Keap1-Nrf2 PPI inhibitors that are potential candidates for oxidative stress-mediated diseases treatment.

## Declaration of competing interest

None.

## Appendix A Supplementary data

Materials and Methods were reported in the Supporting information. Keap1 and Nrf2 protein expression and purification were showed in Supplement Figure 1. Selected chemicals for biological activity test was showed in Supplement Figure 2. The impact of three drugs on Nrf2 nuclear translocation was showed in Supplement Figure 3. The impact of three FDA-approved drugs on H_2_O_2_-induced cell death and apoptosis in both SH-SY5Y cells and PC12 cells was showed in Supplement Figure 4. The impact of three FDA-approved drugs on LPS-challenged mice was showed in Supplement Figure 5. The docking parameter validation was showed in Supplement Figure 6. Top 20 candidates filtered as potential PPI inhibitors of Keap1−Nrf2 from FDA-approved drugs was showed in Supplement Table 1. Original indications of three FDA-approved drugs was summarized in Supplement Table 2. Animal experiment design was demonstrated in Supplement Table 3. The advantages and disadvantages of Keap1-Nrf2 inhibitor screening assay were analyzed in Supplement Table 4.
