# Supplementary material for: Development of an enzyme-linked immunosorbent assay for Keap1-Nrf2 interaction inhibitors identification
Source: Redox Biol. 2020 May 12;34:101573. doi: 10.1016/j.redox.2020.101573 (PMC7231848; doi:10.1016/j.redox.2020.101573)
Supplement: Multimedia component 1 [file mmc1.docx]

Submitted to Redox Biology

Supplementary data

**Development of an Enzyme-linked Immunosorbent Assay for Keap1-Nrf2 Interaction Inhibitors Identification**

Yan Wang^†,‡,$,^*, Chu-Ying Xiao^†,$^, Huang-Quan Lin^†,#^, Jian-Shu Hu^†^, Tsz-Ming Ip^†^ and David Chi-Cheong Wan^†,^*

^†^ School of Biomedical Sciences, Faculty of Medicine, The Chinese University of Hong Kong, Shatin, Hong Kong SAR, China

^‡^ Center for Translation Medicine Research and Development, Institute of Biomedical and Health Engineering, Shenzhen Institute of Advanced Technology, The Chinese Academy of Sciences, Shenzhen 518055, China

^#^ Shenzhen Research Institute, the Chinese University of Hong Kong, Shenzhen 518057, China

^*^ Corresponding author

E-mail addresses: [yan.wang@siat.ac.cn](mailto:yan.wang@siat.ac.cn) (Y. Wang)

Postal address: 1068 Xueyuan Avenue, Shenzhen University Town, Nanshan, Shenzhen, P.R.China

E-mail addresses: [chicheongwan@e.cuhk.edu.hk](mailto:chicheongwan@e.cuhk.edu.hk) (D.C.C. Wan)

Postal address: 326A, Lo Kwee-Seong Integrated Biomedical Sciences Building, Area39, The Chinese University of Hong Kong, Shatin, N.T., Hong Kong SAR, P. R.China.

^$^ Y.W., and C.-Y.X. contributed equally to this work.

**Index**

**Materials and Methods**∙∙∙∙∙∙∙∙∙∙∙∙∙∙∙∙∙∙∙∙∙∙∙∙∙∙∙∙∙∙∙∙∙∙∙∙∙∙∙∙∙∙∙∙∙∙∙∙∙∙∙∙∙∙∙∙∙∙∙∙∙∙∙∙∙∙∙∙∙∙∙∙∙∙∙∙∙∙∙∙∙∙∙∙∙∙∙∙∙∙∙∙∙∙**3-11**

**Figure S1. Keap1 and Nrf2 protein expression and purification**∙∙∙∙∙∙∙∙∙∙∙∙∙∙∙∙∙∙∙∙∙∙∙∙∙∙∙∙**12**

**Figure S2. Selected chemicals for biological activity test**∙∙∙∙∙∙∙∙∙∙∙∙∙∙∙∙∙∙∙∙∙∙∙∙∙∙∙∙∙∙∙∙∙∙∙∙∙∙∙∙∙**13**

**Figure S3. Three FDA-approved drugs induce Nrf2 nuclear translocation in both SH-SY5Y cells and PC12 cells** ∙∙∙∙∙∙∙∙∙∙∙∙∙∙∙∙∙∙∙∙∙∙∙∙∙∙∙∙∙∙∙∙∙∙∙∙∙∙∙∙∙∙∙∙∙∙∙∙∙∙∙∙∙∙∙∙∙∙∙∙∙∙∙∙∙∙∙∙∙∙∙∙∙∙∙∙∙∙∙∙∙∙∙∙**14**

**Figure S4. Three FDA-approved drugs attenuate H_2_O_2_-induced cell death and apoptosis in both SH-SY5Y cells and PC12 cells** ∙∙∙∙∙∙∙∙∙∙∙∙∙∙∙∙∙∙∙∙∙∙∙∙∙∙∙∙∙∙∙∙∙∙∙∙∙∙∙∙∙∙∙∙∙∙∙∙∙∙∙∙∙**15**

**Figure S5. Three FDA-approved drugs attenuate lipopolysaccharide (LPS)-induced inflammation in mice**∙∙∙∙∙∙∙∙∙∙∙∙∙∙∙∙∙∙∙∙∙∙∙∙∙∙∙∙∙∙∙∙∙∙∙∙∙∙∙∙∙∙∙∙∙∙∙∙∙∙∙∙∙∙∙∙∙∙∙∙∙∙∙∙∙∙∙∙∙∙∙∙∙∙**16**

**Figure S6. Docking parameter validation**∙∙∙∙∙∙∙∙∙∙∙∙∙∙∙∙∙∙∙∙∙∙∙∙∙∙∙∙∙∙∙∙∙∙∙∙∙∙∙∙∙∙∙∙∙∙∙∙∙∙∙∙∙∙∙∙∙∙∙∙∙∙∙∙∙∙**17**

**Table S1. Top 20 candidates filtered as potential PPI inhibitors of Keap1−Nrf2 from FDA-approved drugs**∙∙∙∙∙∙∙∙∙∙∙∙∙∙∙∙∙∙∙∙∙∙∙∙∙∙∙∙∙∙∙∙∙∙∙∙∙∙∙∙∙∙∙∙∙∙∙∙∙∙∙∙∙∙∙∙∙∙∙∙∙∙∙∙∙∙∙∙∙∙∙∙∙∙∙∙∙∙∙∙∙∙∙∙∙∙∙∙∙∙∙**18**

**Table S2. Original indications of three FDA-approved drugs**∙∙∙∙∙∙∙∙∙∙∙∙∙∙∙∙∙∙∙∙∙∙∙∙∙∙∙∙∙∙∙∙**19**

**Table S3. Animal experiment design**∙∙∙∙∙∙∙∙∙∙∙∙∙∙∙∙∙∙∙∙∙∙∙∙∙∙∙∙∙∙∙∙∙∙∙∙∙∙∙∙∙∙∙∙∙∙∙∙∙∙∙∙∙∙∙∙∙∙∙∙∙∙∙∙∙∙∙∙∙∙∙∙∙∙∙**20**

**Table S4. The analysis of advantages and disadvantages of Keap1-Nrf2 inhibitor screening assay**∙∙∙∙∙∙∙∙∙∙∙∙∙∙∙∙∙∙∙∙∙∙∙∙∙∙∙∙∙∙∙∙∙∙∙∙∙∙∙∙∙∙∙∙∙∙∙∙∙∙∙∙∙∙∙∙∙∙∙∙∙∙∙∙∙∙∙∙∙∙∙∙∙∙∙∙∙∙∙∙∙∙∙∙∙∙∙∙∙∙∙∙∙∙∙∙∙∙∙∙∙∙∙∙∙∙∙∙∙∙∙**21**

**Materials and Methods**

**Molecular Docking**

The Keap1 structures were downloaded from the RCSB Protein Data Bank (PDB, <http://www.rcsb.org/pdb/home/home.do>), including PDB ID code 47LB, 47LC, 47LD, 4N1B, 4IN4, and 4IQK. All of the PDB files were firstly processed by Chimera (UCSF Resource for Biocomputing, Visualization, and Informatics, CA, USA) to delete the ligand and the water molecule by AutoDock Tools (The Scripps Research Institute, CA, USA) to add hydrogens, add Gasteiger charge, and assign AD4 type on atoms making them recognized, and as a result the PDB files are converted to PDBQT files that available as a receptor for molecular docking screening to find out the inhibitor of Keap1-Nrf2 PPIs. The chemicals from original protein models were selected as positive controls (benchmark) to validate our docking protocol. Our docking simulation showed that the predicted conformations of the inhibitors are close to the experimental conformations of the inhibitors (Figure S6). Furthermore, the inhibitor exhibited a high binding score against Keap1. The FDA approved drug data containing ~1500 drugs were compiled by our previously described method (Y. Wang et al., 2014). Each of the ligand was converted to PDBQT file by AutoDock Tool (The Scripps Research Institute, CA, USA). The binding site where the ligand insert into the receptor is defined “grid box” in the AutoDock Tool. The grid box setting depended on the structure of binding pocket of the receptor as 20Å×20Å×20Å, which encompassing the whole structure of ligand. The AutoDock Vina (The Scripps Research Institute, CA, USA) was used for screening. The docking result of receptor-ligand binding were generated by the program and further analyzed by PyMOL. Moreover, the program also showed the docking score, which revealed the binding affinity of each ligand and receptor. The relationship between score and binding were analyzed by PyMOL directly. Finally the docking score of ~1500 drugs were listed for further exploration.

**Reagents and antibodies**

The Keap1 (E-20) and Nrf2 (C-20) gene specific antibodies were purchased from *Santa Cruz Biotech*. The streptavidin conjugated alkaline phosphatase was from *USB Products Affymetrix*. The secondary antibodies were purchased from ZYMED *Thermo Fisher Scientific*. The selected FDA-approved drugs, zafirlukase, dutasteride, gliquidone, ketoconazole, drospirenone, indinavir and saquinavir, the positive control DMF and the pNPP tablets were form *Sigma*. Seven test chemicals were dissolved in distilled water and dimethyl sulfoxide (DMSO); the final concentration of DMSO was less than 0.1%.

**Plasmid construction**

Full length human Nrf2 in pcDNA3-Myc3 plasmid was double digested overnight by restriction enzymes BamH I and Kpn I. The primers designed for PCR are as followed: forward primer GACGACGGATCCATGGATTTGATTGACATACTT, and reverse primer TGATGCGGTACCCTAGTTTTTCTTAACATCTGG. The PCR product and pRSET-b vector were incubated with T4 ligase overnight. The ligation product was transformed into *E. coli* DH5α. After DNA purification and concentration, the recombinant DNA containing a his-tag at N terminal of Nrf2 was confirmed by sequencing. Full length human Keap1 in pDsRed2 plasmid were double digested overnight by restriction enzyme Nde I and Kpn I. The primers designed for PCR are as followed: forward primer AAAGGGCATATGCAGCCAGATCCCAGGCCT, and reverse primer GACCAGCAGAACTGTACCTGTGGTACCATCATC. The PCR product and PCOLADuet-1 vector were incubated with T4 ligase overnight. The ligation product was transformed into *E. coli* *DH5α*. After DNA purification and concentration, the recombinant DNA containing a BirA gene (the biotin ligase) with a his-tag and the Keap1 gene with an Avi-tag (biotin recognition site) was confirmed by sequencing.

**Protein expression and purification**

The his-tag Nrf2 in pRSET-b vector and the Avi-tag Keap1 PCOLADuet-1 vector were transformed in *E. coli* BL21 (DE3) pLysS, respectively for protein expression. A 250 ml bacterial culture was allowed to grow at 37°C till the growth phase (OD 600 nm was 0.4 to 0.6), then 0.5 mM IPTG was added to the culture and incubated for 16 h at 37°C. In order to link Keap1 with biotin, 50 mM biotin was added to the bacterial culture together with IPTG when inducing the Keap1 and birA expression. The induced bacterial culture was harvested by centrifuged 5000 rpm for 30 min at 4°C. The pellet was re-suspended by column 25 ml binding buffer (20 mM Tris-HCl pH 7.8, 500 mM NaCl, containing 5 mM imidazole) containing protease inhibitor cocktail. The cells were sonicated for 30 min and centrifuged at 20, 000 rpm for 30 min at 4°C. The supernatant was filtered by a 0.2 μM filter and applied to the 3-cm nickel column equilibrated with binding buffer. For his-tag Nrf2 purification, 10 ml binding buffer was applied to the column to remove most of the bacterial proteins; 20 ml washing buffer (20 mM Tris-HCl pH 7.8, 500 mM NaCl, containing 40 mM imidazole) was applied to wash the his-tag Nrf2 and Nrf2 was eluted by 10 ml elute buffer (60 mM Tris-HCl pH 7.8, 500 mM NaCl, containing 200 mM imidazole). For Avi-tag Keap1 protein purification, 10 ml binding buffer was applied to the column to remove most of the bacterial proteins; 20 ml washing buffer (20 mM Tris-HCl pH 7.8, 500 mM NaCl, containing 20 mM imidazole) was applied to wash the Avi-tag Keap1 and Keap1 was eluted by 10 ml elute buffer (20 mM Tris-HCl pH 7.8, 500 mM NaCl, containing 80 mM imidazole). The proteins were concentrated by Amicon ultra-15 centrifuge filter devices. The purified Nrf2 protein was identified by Nrf2 specific antibody while purified Keap1 protein was identified by streptavidin conjugated alkaline phosphatase and Keap1 specific antibody, respectively. The proteins concentration was determined by NanoDrop 200 Protein A_280_.

**ELISA format assay**

To determine the binding ability of biotinylated Keap1 against the streptavidin-coated plate, serial dilution of biotinylated Keap1 was immobilized on the streptavidin-conjugated plate. The plate was incubated at room temperature for 30 min and washed three times with plate washing buffer. The streptavidin-binding Keap1 was quantified by streptavidin conjugated alkaline phosphatase. To determine the binding ability of Keap1 against Nrf2, serial dilution of his-tag Nrf2 was incubated with Keap1 for 30 min and washed three times with plate washing buffer. The Keap1-binding Nrf2 was probed by Nrf2 antibody and quantified by alkaline phosphatase conjugated secondary antibody. The inhibitory effect of DMF and other FDA-approved drugs on Keap1-Nrf2 interaction was analyzed by ELISA format assay. The percentage inhibitory activities of the various compounds were calculated by comparison with the positive control and the blank control. The formula was shown as follows: percent of inhibitory activity of the compound = 1 – (absorbance of sample –absorbance of blank control)/(absorbance of blank control – absorbance of blank control) × 100%. Data analysis was performed with Prism software. Inhibitory effects were expressed as IC_50_ value calculated by regression analysis.

**FP competition assays**

A competition assay using the conditions described in manufacture's manual (the KEAP1-Nrf2 Inhibitor Screening Assay Kit, BPS Bioscience, USA) was used to determine the potency of inhibitors of Keap1-Nrf2 interaction. Each well had a final volume of 50 μL that consisted of 0.5 μL of 1 μM FITC-9mer Nrf2 peptide amide and 20 μL of 15ng/μL Keap1 Kelch domain protein, 24 μL of assay buffer, and 5 μL of an inhibitor sample of varying concentrations. The binding experiments were performed in triplicates with initial concentration of the inhibitor typically set. Read the fluorescent polarization of the sample in a microtiter-plate reader capable of excitation at wavelengths ranging from 475-495 nm and detection of emitted light ranging from 518-538 nm. The percentage inhibitory activities of the various compounds were calculated by comparison with the positive control and the blank control. The formula was shown as follows: percent of inhibitory activity of the compound = 1 – (absorbance of sample –absorbance of blank control)/(absorbance of blank control – absorbance of blank control) × 100%. Data analysis was performed with Prism software. Inhibitory effects were expressed as IC_50_ value calculated by regression analysis.

**Cell culture**

Rat adrenal medulla pheochromocytoma PC12 cells were from ATCC (Manassas, VA, USA) and seeded in the DMEM medium containing 10% FBS at 37°C with 95% humidified air and 5% CO_2_. Human neuroblastoma SH-SY5Y cells were from ATCC (Manassas, VA, USA) and seeded in the F12/DMEM medium containing 10% FBS at 37°C with 95% humidified air and 5% CO_2_. The cells were passaged by 1:4 for further experiments.

**Cell viability**

The cell viability assay was determined by MTT assay. Cells were digested by trypsin for 1 minute to make the suspension, and were seeded in 96-well plate and the density of cells was calculated as 5×10^3^ cells/well. And then the cells were treated with selected drugs with the concentration at 10 μM at 37°C for 24 hours. After that cells were treated with 250 μM H_2_O_2_ for 4 h. Subsequently, cells were treated with 5mg/mL MTT 20 μL for 4 h, and 150 μL DMSO was added to elute the cell. The results were given by the spectrophotometer (Ultramark Microplate Reader, Bio-Rad) at the 590 nm.

**Cytoplasm and nuclear protein preparation**

Cells were washed with PBS and incubated in cell lysis buffer [10 mM HEPES; pH 7.5, 10 mM KCl, 0.1 mM EDTA, 1 mM DTT, 0.5% NP-40 and 0.5 mM PMSF] on ice for 15 min and homogenized. The supernatant containing cytoplasm proteins were collected after 15 min 12,000 g centrifugation. Nuclei were washed once using cell lysis buffer and extracted for 30 min on ice in nuclear extraction buffer [20 mM HEPES (pH 7.5), 420 mM NaCl, 1 mM EDTA, 1 mM DTT and 1 mM PMSF]. Insoluble material was removed by ultracentrifugation for 10 min. The supernatant was used as the nuclear extract.

**Western blot**

Proteins in the cytoplasm and nuclear were separated by 10% SDS polyacrylamide gel electrophoresis and electrotransferred to a polyvinylidene difluoride membrane (Immobilon-P membrane; Millipore, Bedford, MA, USA). After the blot was blocked in a solution of 5% bovine serum albumin, membrane was incubated overnight with primary antibodies against Nrf2 and β-actin followed by incubation with alkaline phosphatase conjugated secondary antibodies for 1 h. Specific bands were detected with BCIP/NBT western blotting detection reagent (GE Healthcare Bio-Sciences).

**RNA isolation and quantitative real-time PCR**

RNA isolation was performed according to previous study. HO-1, NQO1 and Nrf2 messenger RNA (mRNA) expression were determined by use of the Applied Biosystems ViiA™ 7 Real Time PCR System (Applied Biosystems, Foster City, CA, USA). The real-time RT-PCR in a volume of 10 μL were performed in triplicate under the following conditions: The PCR mixture was set up in a 20-μL reaction containing 1 μL of the cDNA as the template and 0.5 μL of each 10 μM forward primer and 10 μM reverse primer and 5 μl 2× Power SYBR Green PCR master mix, and the volume was adjusted by autoclaved distilled water. The primers of HO-1 (forward, 5ʹ-GGGTGATAGAAGAGGCCAAGA-3ʹ; reverse, 5ʹ-AGCTCCTGCAACTCCTCAAA-3ʹ), Nrf2 (forward, 5ʹ-CTCGCTGGAAAAAGAAGTG-3ʹ; reverse, 5ʹ-CCGTCCAGGAGTTCAGAGG-3ʹ), NQO1 (forward, 5’- AGCGCTTGACACTACGATCC-3’; reverse, 5’-CAATCAGGGCTCTTCTCACC-3’), and GAPDH (forward, 5′-TATGATGATATCAAGAGGGTAGT-3′, reverse, 5′-TGTATCCAAACTCATTGTCATAC-3′) were synthesized by Life technologies (Hong Kong, Hong Kong).

**Animal study**

Female C57BL/6 mice (12–16 weeks) were housed in a temperature-controlled room (22°C) with a 12-h light/dark cycle and received food and water ad libitum. The mice were divided into 9 groups, 10 mice each group. 3 groups for control experiments (blank control, dexamehasone 10 mg/kg/day, lipopolysaccharide (LPS) 2 mg/kg) and 6 groups for testing experiments (Low dose 10 mg/kg/day and high dose 40 mg/kg/day of Keap1-Nrf2 PPI inhibitors from FDA-Approved Drug Database, respectively). The mice (except the blank control group and LPS group) were treated with dexamethasone or Keap1-Nrf2 PPI inhibitors by intragastric administration for 5 days (day 1, 2, 3, 4 and 5) beginning at 12-16 weeks old. All mice (except the blank control group) were challenged with LPS by intraperitoneal injection at day 5. 5h after LPS challenging, all mice were sacrifised by overdose anesthesia and blood were collected. Indivifual blood samples (n=10) were left undisturbed at room temperature for 30 min. Clot was removed by refrigerated centrifuge at 10000 rpm for 15 min and serum samples were collected. Cytokines (TNF-α, IL-6 and IL-12 p70 ) in serum samples were measured by ELISA kit (CSB-E04741m, Mouse Tumor necrosis factor α,TNF-α ELISA KIT, CUSABIO, China, CSB-E04600m, Mouse Interleukin 12,IL-12/P70 ELISA KIT, CUSABIO, China and CSB-E04639m, Mouse Interleukin 6,IL-6 ELISA KIT, CUSABIO, China).


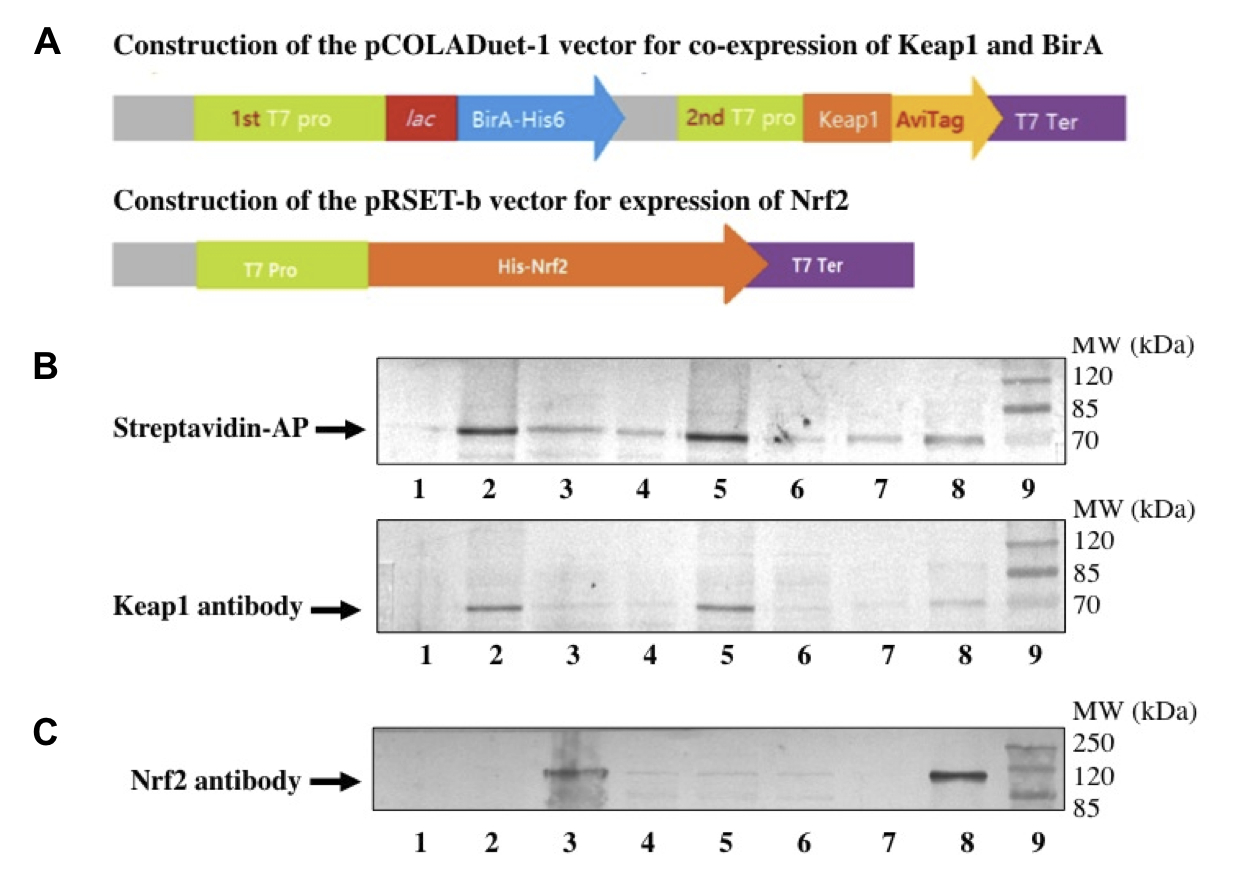


**Figure S1. Keap1 and Nrf2 protein expression and purification.** (A) The construction of the pCOLADuet-1 vector for co-expression of Keap1 and BirA ligase was illustrated in the top panel, whereas the construction of the pRSET-b vector for expression of Nrf2 was illustrated in the bottom panel. (B) Western blot analysis of Keap1 protein expression and purification using streptavidin-AP and specific Keap1 antibody, respectively. Line 1: empty vector whole bacterial lysate, line 2: induced Keap1 whole bacterial lysate, line 3: non-induced Keap1 whole bacterial lysate, line4: pellet, line 5: supernatant, line 6: NTA column flow out, line 7: washing fraction, line 8: elution, line 9: the ladder. (C) Western blot analysis of Nrf2 protein expression and purification using specific Nrf2 antibody. Line 1: empty vector whole bacterial lysate, line 2: non-induced Nrf2 whole bacterial lysate, line 3: supernatant, line 4: pellet, line 5: NTA column flow out, line 6: binding fraction, line 7: washing fraction, line 8: elution, line 9: the ladder.

**
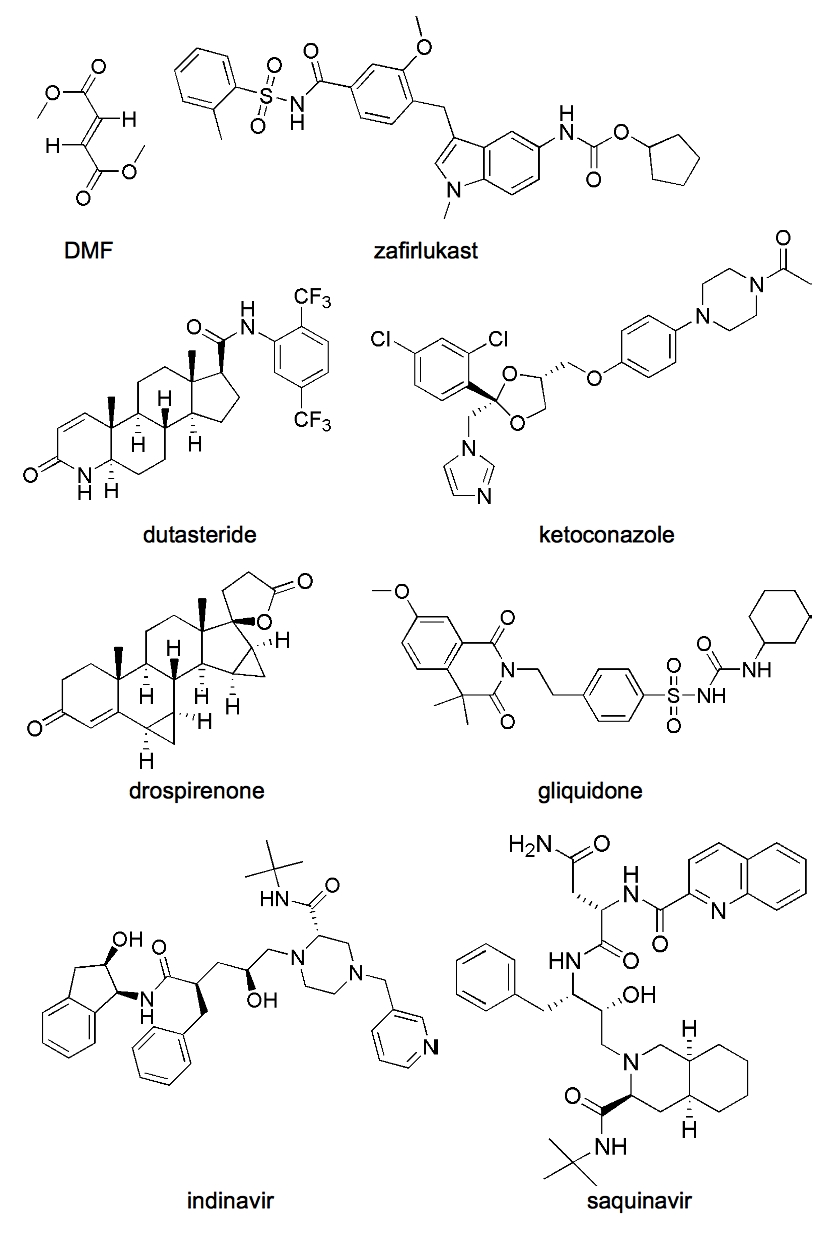
**

**Figure S2. Selected chemicals for biological activity test**

**
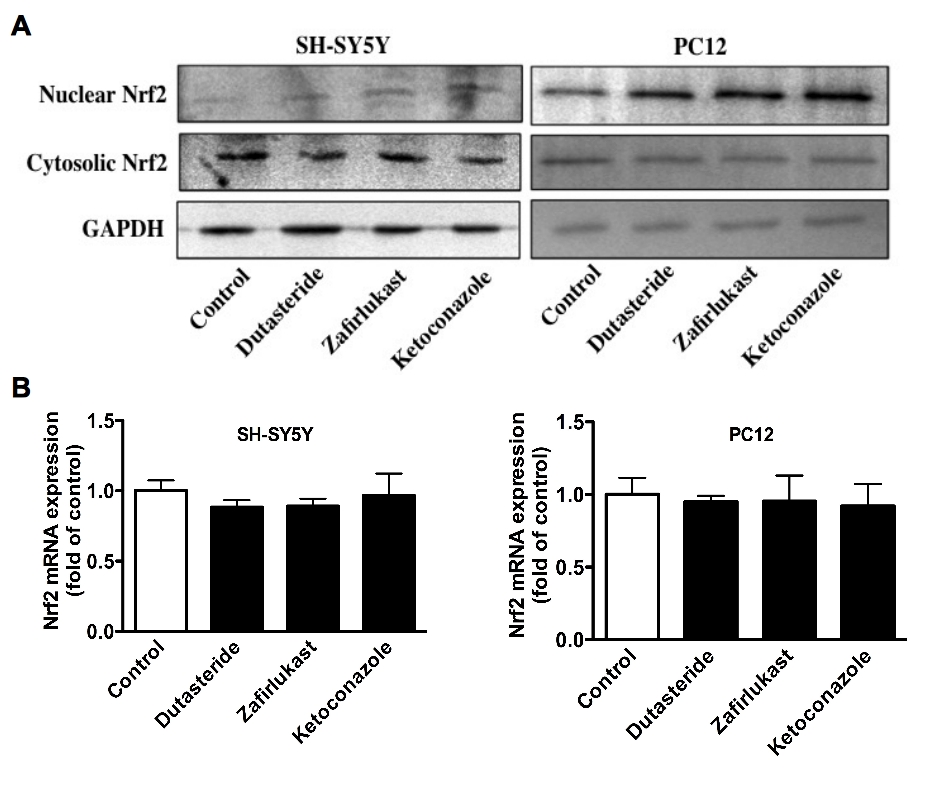
**

**Figure S3. Three FDA-approved drugs induce Nrf2 nuclear translocation in both SH-SY5Y cells and PC12 cells.** (A) Western blot analysis. SH-SY5Y cells or PC12 cells were treated with DMSO or test drugs (10 μM) for 6 hours and cytoplasmic and nuclear extracts were separated and immunoblotted with anti-Nrf2, and anti-GAPDH antibodies. (B) After 4 hours treatment, the cells were harvested, the total mRNA was collected for reverse transcription, and quantitative PCR measured. Results were repeated at least three times from three independent experiments and presented as mean ± SD, n = 3. The results were statistically analyzed by one-way ANOVA test.

**
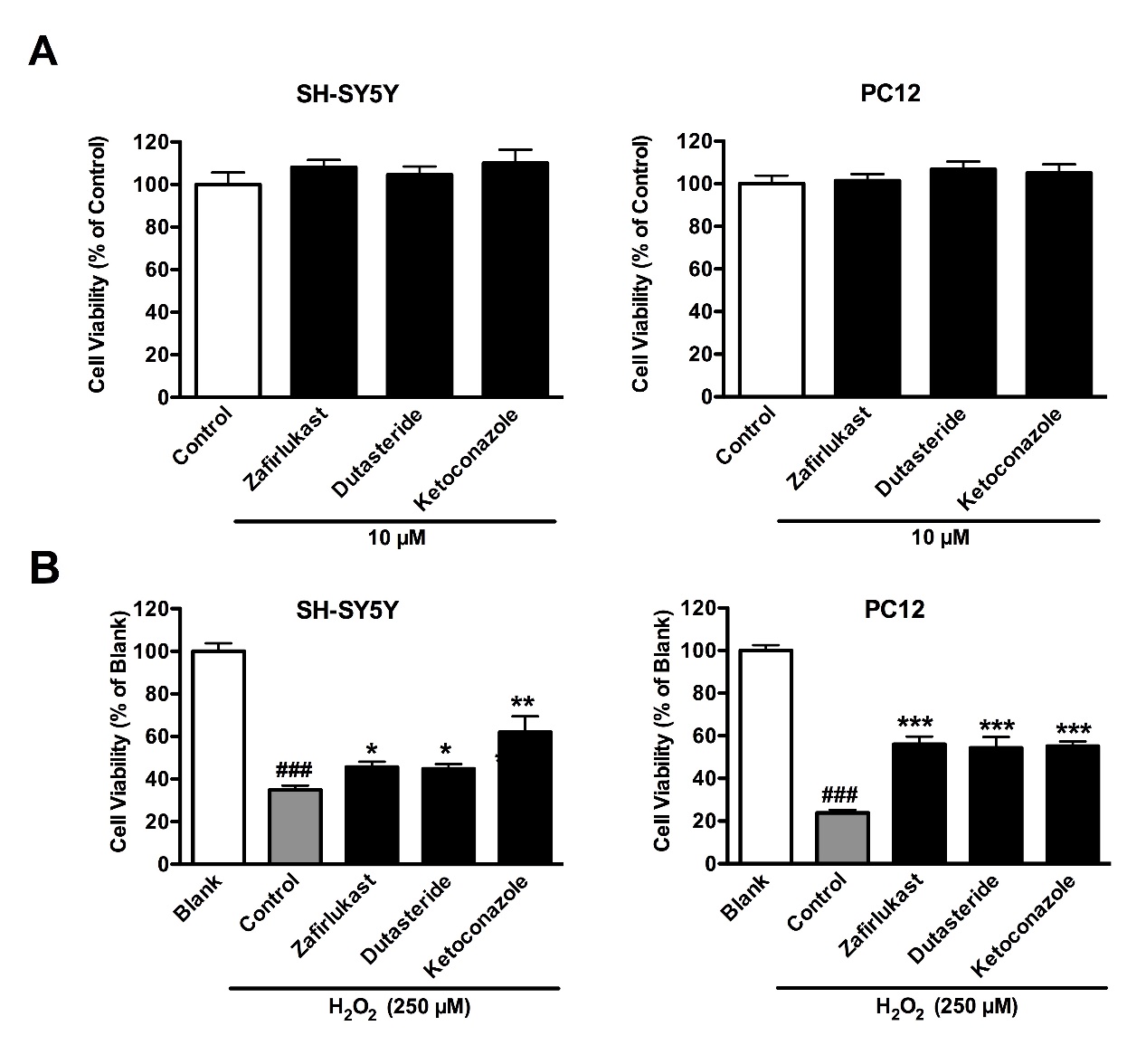
Figure S4. Three FDA-approved drugs attenuate H_2_O_2_-induced cell death and apoptosis in both SH-SY5Y cells and PC12 cells.** (A) For cytotoxicity test, SH-SY5Y cells or PC12 cells were incubated with different potential Keap1-Nrf2 inhibitors (10 μM) for 24 hours, and the viability was detected by MTT. Data shown are means ± SD of results from independent experiments in triplicate. (B) SH-SY5Y cells or PC12 cells were incubated with different potential Keap1-Nrf2 inhibitors (10 μM) for 12 hours and then stimulated with H_2_O_2_ (250 μM) for 4 hours, and the cell viability was measured by MTT assay. Data shown are means ± SD of results from independent experiments in triplicate. ^###^P < 0.005 compared with control cells; ^*^P < 0.05 compared with H_2_O_2_-stimulated cells; ^**^P < 0.01 compared with H_2_O_2_-stimulated cells; ^***^P < 0.005 compared with H_2_O_2_-stimulated cells.


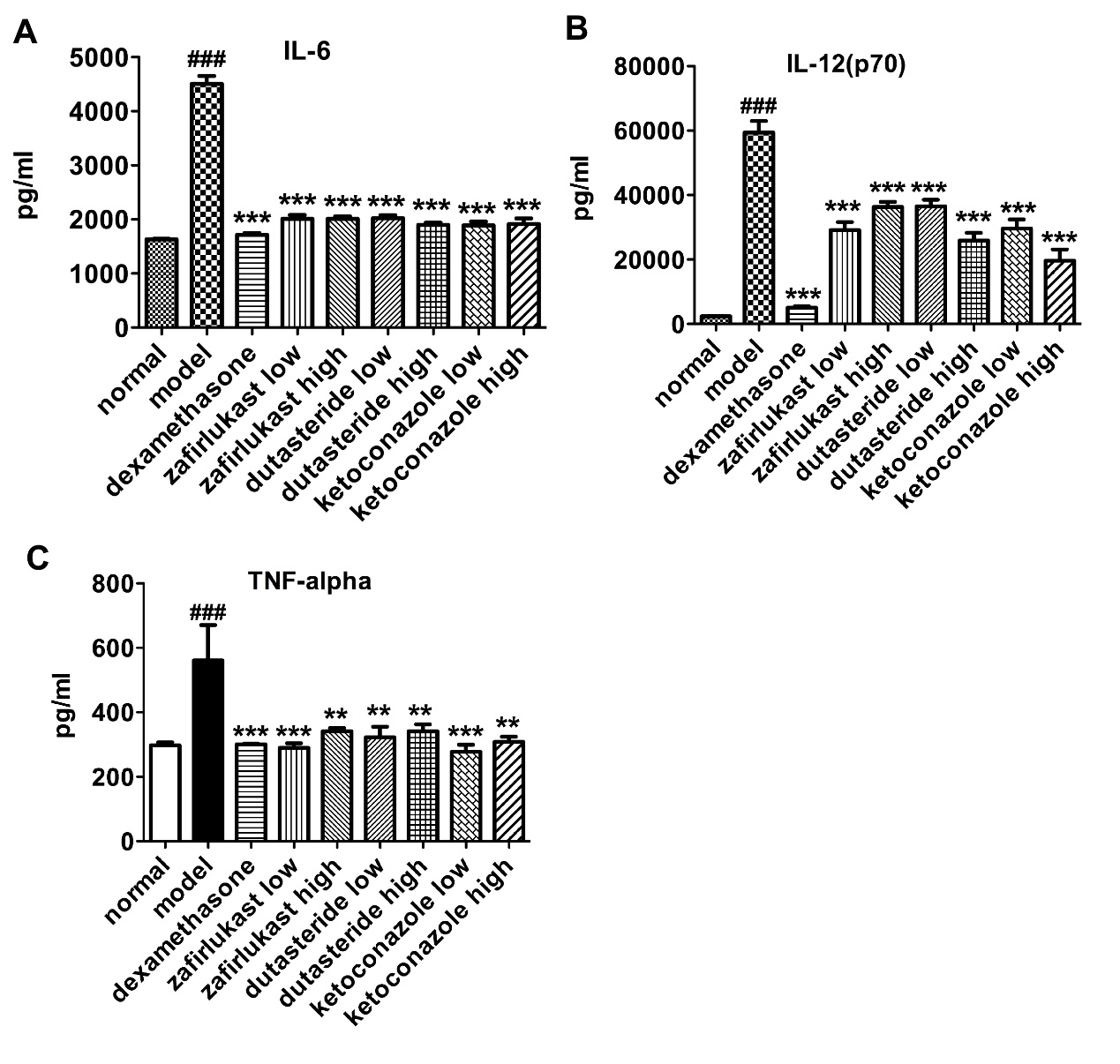
**Figure S5. Three FDA-approved drugs attenuate lipopolysaccharide (LPS)-induced inflammation in** **mice.** Three groups for control experiments (blank control, dexamehasone 10 mg/kg/day, lipopolysaccharide (LPS) 2 mg/kg) and six groups for testing experiments (Low dose 10 mg/kg/day and high dose 40 mg/kg/day of Keap1-Nrf2 PPI inhibitors from FDA-Approved Drug Database, respectively). The mice (except the blank control group and LPS group) were treated with dexamethasone or Keap1-Nrf2 PPI inhibitors by intragastric administration for 5 days (day 1, 2, 3, 4 and 5) beginning at 12-16 weeks old. All mice (except the blank control group) were challenged with LPS by intraperitoneal injection at day 5. 5h after LPS challenging, all mice were sacrifised by overdose anesthesia and blood were collected. IL-6 (A), IL-12 p70 (B) and TNF-α (C) in serum samples were measured by ELISA kit. ^###^P < 0.005 compared with normal group; ^*^P < 0.05 compared with LPS-stimulated group; ^**^P < 0.01 compared with LPS-stimulated group; ^***^P < 0.005 compared with LPS-stimulated group.


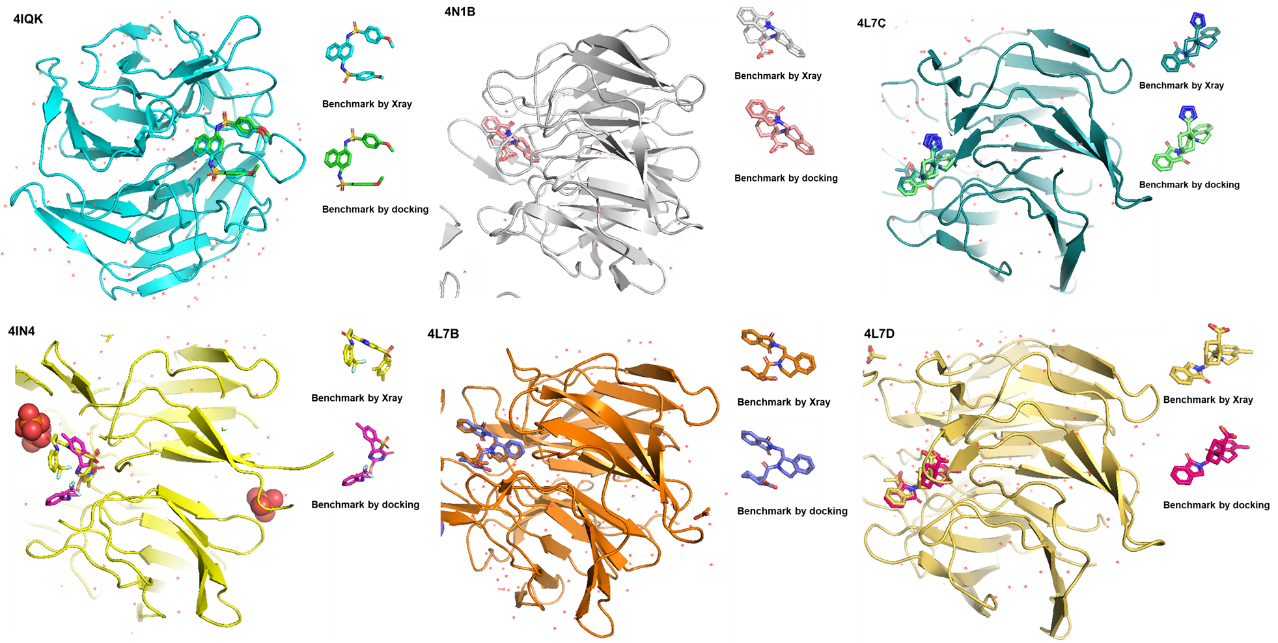


**Figure S6. Molecular docking analysis illustrates the favorable binding positions of benchmark (positive control) with lowest binding free energy.** The three-dimensional diagram shows the interactions of benchmark (colored stick) to Keap1 (colored cartoon). Docking score of benchmark in its corresponding model: 1W (4L7B benchmark, -10.8 kcal/mol); 1VW (4L7C benchmark, -10.9 kcal/mol); 1VX (4L7D benchmark, -11.3 kcal/mol); 2FS (4N1B benchmark, -11.4 kcal/mol); 4ID (4IN4 benchmark, -10.3 kcal/mol); IQK (4IQK benchmark, -10.6 kcal/mol).

**Table S1. Top 20 candidates filtered as potential PPI inhibitors of Keap1−Nrf2 from FDA-approved drugs**

| **Ranking** | **Ligand** | **Docking score (kcal/mol)** | | | | | | |
| --- | --- | --- | --- | --- | --- | --- | --- | --- |
|  |  | **4L7B** | **4L7C** | **4L7D** | **4N1B** | **4IN4** | **4IQK** | **Average** |
| 1 | **Zafirlukast** | -11.2 | -10.7 | -10.1 | -10.8 | -10.5 | -10.2 | -10.58 |
| 2 | **Dutasteride** | -10.8 | -10.7 | -10.5 | -10.1 | -10.5 | -10.3 | -10.48 |
| 3 | Rifapentine | -10.7 | -9.4 | -10.9 | -10.6 | -10 | -10.4 | -10.33 |
| 4 | Ergotamine_C | -10.8 | -10.5 | -10.7 | -10 | -9.7 | -10.2 | -10.32 |
| 5 | Irinotecan | -9.6 | -9.7 | -9.4 | -10.3 | -11.1 | -11.2 | -10.22 |
| 6 | Lapatinib | -10.8 | -9.3 | -9.5 | -10.4 | -10.6 | -10.6 | -10.20 |
| 7 | **Gliquidone** | -10.5 | -9.8 | -9.7 | -9.9 | -10.4 | -10.7 | -10.17 |
| 8 | **Ketoconazole** | -10.4 | -9.4 | -9.0 | -10.8 | -10.2 | -11.2 | -10.17 |
| 9 | Antrafenine | -10.1 | -9.8 | -8.9 | -10.5 | -10.5 | -11 | -10.13 |
| 10 | Adapalene | -10.3 | -10.3 | -9.9 | -10.1 | -9.5 | -10.4 | -10.08 |
| 11 | Dihydroergotamine | -10.1 | -9.3 | -10.2 | -10.8 | -10.2 | -9.8 | -10.07 |
| 12 | Imatinib | -11 | -8.9 | -8.7 | -10.6 | -10.7 | -10.3 | -10.03 |
| 13 | **Drospirenone** | -10.7 | -10.3 | -9.9 | -9.4 | -10 | -9.6 | -9.98 |
| 14 | **Indinavir** | -9.7 | -9.9 | -9.0 | -10.3 | -10.7 | -10.2 | -9.97 |
| 15 | **Saquinavir** | -8.7 | -9.7 | -10.1 | -10.2 | -11.3 | -9.8 | -9.97 |
| 16 | Tadalafil | -10.1 | -10.1 | -10.0 | -9.5 | -10.4 | -9.5 | -9.93 |
| 17 | Teniposide_C | -10.2 | -10.1 | -9.2 | -10 | -10.1 | -10 | -9.93 |
| 18 | Terconazole | -10.5 | -8.6 | -8.8 | -10.4 | -10.2 | -11.1 | -9.93 |
| 19 | Paliperidone | -9.9 | -9.3 | -8.9 | -10.5 | -10.6 | -10.2 | -9.90 |
| 20 | Ergotamine_Iso | -9.7 | -10.0 | -9.8 | -10.1 | -10 | -9.7 | -9.88 |

Docking score in red indicates that this score is lower than benchmark’s score. Docking score of benchmark in its corresponding model: 1W (4L7B benchmark, -10.8 kcal/mol); 1VW (4L7C benchmark, -10.9 kcal/mol); 1VX (4L7D benchmark, -11.3 kcal/mol); 2FS (4N1B benchmark, -11.4 kcal/mol); 4ID (4IN4 benchmark, -10.3 kcal/mol); IQK (4IQK benchmark, -10.6 kcal/mol). The selected drugs for the biological evaluation are highlighted in bold font.

**Table S2. Original indications of three FDA-approved drugs**

| **FDA-approved drug** | **Indication** | **Antioxidant effects** |
| --- | --- | --- |
| Zafirlukast | Chronic treatment of asthma | Exhibit antioxidant effects in human cells (Al-Zamil HA, et al. *Curr Ther Res Clin Exp*. 2005;66(4):279-93.) |
| Dutasteride | Symptomatic benign prostatic hyperplasia | No report |
| Ketoconazole | Fungal infections | Exhibit antioxidant effects in rat liver microsomes (Wiseman H, et al. *Chem Biol Interact*. 1991;79(2):229-43.) |

**Table S3. Animal experiment design**

|  | **Blank Control** | **Model Control** | **Positive Control (Dexamehasone)** | **Keap-Nrf2 interaction inhibitors** | | | | | |
| --- | --- | --- | --- | --- | --- | --- | --- | --- | --- |
|  |  |  |  | **zafirlukase** | | **dutasteride** | | **ketoconazole** | |
| Lipopolysaccharide (LPS, mg/kg) | 0 | 2 | 2 | 2 | 2 | 2 | 2 | 2 | 2 |
| Test Drug (mg/kg/day) | 0 | 0 | 10 | 10 | 40 | 10 | 40 | 10 | 40 |

**Table S4. The analysis of advantages and disadvantages of Keap1-Nrf2 inhibitor screening assay**

|  | **ELISA** | **FP** | **FRET** |
| --- | --- | --- | --- |
| Adopt for high-throughput assay | No | Yes | Yes |
| High background noise | No | Yes | Yes |
| Influenced by autofluorescence of test compounds | No | Yes | Yes |
| Identify Keap1-ETGE binding inhibitor | Yes | Yes | Yes |
| Identify Keap1-DLG binding inhibitor | Yes | No | No |
| Cost | Medium | High | Low |
